# Automatic projection image registration for nanoscale X-ray tomographic reconstruction

**DOI:** 10.1107/S1600577518013929

**Published:** 2018-10-23

**Authors:** Haiyan Yu, Sihao Xia, Chenxi Wei, Yuwei Mao, Daniel Larsson, Xianghui Xiao, Piero Pianetta, Young-Sang Yu, Yijin Liu

**Affiliations:** a College of Mechanical Engineering, Donghua University, Shanghai 201620, People’s Republic of China; b Stanford Synchrotron Radiation Lightsource, SLAC National Accelerator Laboratory, Menlo Park, CA 94025, USA; c School of Electronic and Optical Engineering, Nanjing University of Science and Technology, Nanjing, Jiangsu 210094, People’s Republic of China; d School of Computer Science and Technology, Nanjing University of Aeronautics and Astronautics, Nanjing, Jiangsu 211100, People’s Republic of China; e Advanced Photon Source, Argonne National Laboratory, 9700 South Cass Avenue, Lemont, IL 60439, USA; f Advanced Light Source, Lawrence Berkeley National Laboratory, Berkeley, CA 94720, USA

**Keywords:** transmission X-ray microscopy, nanoscale tomography, image registration, tomographic reconstruction

## Abstract

Novel developments in X-ray sources, optics and detectors have significantly advanced the capability of X-ray microscopy at the nanoscale. Depending on the imaging modality and the photon energy, state-of-the-art X-ray microscopes are routinely operated at a spatial resolution of tens of nanometres for hard X-rays or ∼10 nm for soft X-rays. The improvement in spatial resolution, however, has led to challenges in the tomographic reconstruction due to the fact that the imperfections of the mechanical system become clearly detectable in the projection images. Without proper registration of the projection images, a severe point spread function will be introduced into the tomographic reconstructions, causing the reduction of the three-dimensional (3D) spatial resolution as well as the enhancement of image artifacts. Here the development of a method that iteratively performs registration of the experimentally measured projection images to those that are numerically calculated by reprojecting the 3D matrix in the corresponding viewing angles is shown. Multiple algorithms are implemented to conduct the registration, which corrects the translational and/or the rotational errors. A sequence that offers a superior performance is presented and discussed. Going beyond the visual assessment of the reconstruction results, the morphological quantification of a battery electrode particle that has gone through substantial cycling is investigated. The results show that the presented method has led to a better quality tomographic reconstruction, which, subsequently, promotes the fidelity in the quantification of the sample morphology.

## Abstract 2

Automatic alignment of projection images with respect to a common rotation axis is fundamental to the quality of nano-resolution tomographic reconstruction. A hybrid algorithm developed herein demonstrates superior accuracy, efficiency and robustness.

## 1. Introduction   

Ever since the discovery of X-rays in 1895 (Röntgen, 1895 ▸), imaging has been identified as a key area for X-ray applications. While the penetration capability of X-rays facilitates the non-invasive visualization of the specimens’ internal structure, their short wavelength also makes it possible to achieve high spatial resolution that is better than the diffraction limit in conventional light microscopy. When X-ray microscopy is implemented at advanced X-ray facilities, *e.g.* synchrotrons, many different imaging modalities have been demonstrated. With the use of novel X-ray focusing optics (Chang & Sakdinawat, 2014 ▸), *e.g.* Fresnel zone plates, a spatial resolution at tens of nanometres has been demonstrated in both the full-field mode (Andrews *et al.*, 2009 ▸) and scanning mode (Nazaretski *et al.*, 2017 ▸) for hard X-rays. The imaging resolution can be further pushed down to ∼10 nm in the soft X-ray regime (Chao *et al.*, 2005 ▸). Going beyond the real-space imaging, when the coherent property of the incoming X-rays is utilized to encode the structural information into the far-field diffraction patterns (Miao *et al.*, 2015 ▸), the imaging resolution, in principle, can reach to the X-ray wavelength level, although there are other factors, such as the dynamic range of the detector and the scattering power of the sample, that set the practical limit of the spatial resolution for coherent diffractive imaging. Thanks to all the novel developments in X-ray sources, optics, detectors and different imaging modalities (Liu *et al.*, 2013 ▸), high-resolution X-ray microscopy has become very popular and successful. It has opened vast scientific opportunities in many different research fields.

The tomography technique is one of the most important developments in this field as shown by the Nobel Prize in Physiology or Medicine in 1979 (Physiology or Medicine 1979 Press Release, 1979 ▸). In a tomographic scan, three-dimensional (3D) data are acquired through recording the projection images (two-dimensional data) in different view angles (θ). Numerical algorithms (Liu *et al.*, 2007 ▸) are then used to reconstruct the tomographic data into a 3D volume that represents the structure of the sample in the 3D Cartesian space (*x*, *y* and *z* axes). The reconstructed 3D volume is usually subjected to further segmentation (Kaira *et al.* 2018 ▸), visualization and quantification (Liu *et al.*, 2016 ▸), which is often a key to link the imaging data to the functionality of the sample.

In the tomographic data processing pipeline, there are many steps to prepare the projection images before they are fed into the tomographic reconstruction engine. The alignment of the projection images to a common rotation axis (not necessarily the real rotation axis) is a critical step that could greatly affect the quality of the final 3D volume. In a traditional tomography system, we often need to determine the amount of a static offset of the rotation axis with respect to the center column of the projection images (Donath *et al.*, 2006 ▸). The method for such static offset correction can be as simple as trial-and-error (Gürsoy *et al.*, 2014 ▸) or analysis of an image-pair recorded in reverse viewing angles (Yang *et al.*, 2015 ▸); it can also be much more sophisticated involving novel machine-learning algorithms (Yang *et al.*, 2017 ▸). In the case of nanoscale X-ray tomography, the alignment of the projection images becomes more complicated. This is because, when imaging at nanoscale resolution, the mechanical imperfections of the imaging system become clearly detectable, resulting in random jitters of the projection images. Depending on the image acquisition time, the thermal drift of the system could also become an issue that causes further misalignment. Without proper correction to compensate the image jittering, a severe point spread function will be introduced into the reconstructed 3D volume, degrading the reconstructions’ quality significantly.

A class of algorithms based on the concept of ‘tomographic consistency’ (Guizar-Sicairos *et al.*, 2015 ▸) has been proposed and applied to the high-resolution X-ray and electron tomographic reconstructions (Gürsoy *et al.*, 2017 ▸). While excellent reconstruction quality has been shown, there is room for further improvement. In particular, the alignment of the experimentally measured projection images (the target/moving images) with those that are numerically calculated by reprojecting the 3D matrix in the corresponding viewing angles (the reference/fixed images) can be implemented with different image registration algorithms, which all have their own pros and cons. There is often a trade-off between the efficiency and the accuracy that needs to be considered for optimal performance.

Herein, we present a hybrid tomographic image alignment method that involves several image registration algorithms. We systematically evaluate the characteristics of different registration algorithms and proposed a specific sequence that offers the optimal performance in the presented case studies. Going beyond the visual assessment of the reconstruction results, we look into morphological quantification of battery electrode particles that have gone through substantial cycling. Finally, we demonstrated the application of the proposed method to analyzing samples with more significant morphological complexities (*e.g.* a small piece of shale rock). The presented method has led to better quality in the tomographic reconstruction, which, subsequently, promotes the fidelity in the quantification of the sample morphology.

## 2. Results and discussions   

**Figure 1:**
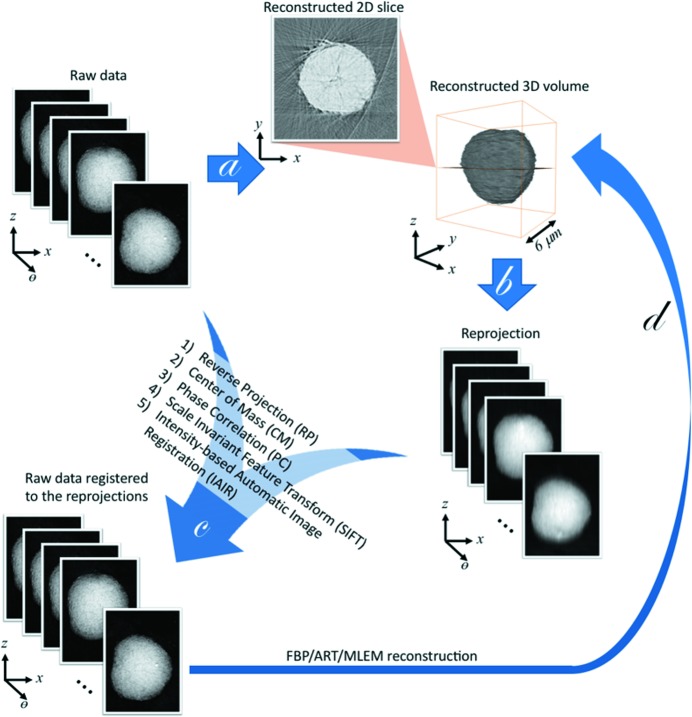
Schematics of the iterative projection image registration workflow for nanoscale X-ray tomographic reconstructions. Step *a* is the initial tomographic reconstruction without registration of the projection images. Step *b* is the reprojection of the 3D matrix in different viewing angles. Step *c* is the registration of the raw data to the reprojected images. As indicated in the inset, several registration algorithms were implemented to conduct step *c*. Step *d* is the tomographic reconstruction of the registered images. Several tomographic reconstruction algorithms are also implemented for this step as well.

Fig. 1 ▸ shows the hybrid tomographic image alignment workflow. After acquiring the two-dimensional projection dataset, the projections are used to reconstruct a first, rough 3D representation of the object (step *a*). The reconstructed 3D volume is converted to a set of calculated projection images (step *b*). Following image registration steps (step *c*) are comparisons between the calculated projection images (fixed reference images) and the original projections (moving images). The resulting set of aligned projections is used as an input for the next iteration (step *d*), where the new calculated projection images are again compared with the originals for alignment. In the early iterations, the challenge originates from the fact that the experimentally measured projection images and the numerically reprojected images are very different in their image quality. The goal of the first few iterations is, therefore, to efficiently center the experimental projection images, whereas the accuracy is not the focus at this stage because the reprojected reference images are of poor resolution anyway. As the iteration continues, we expect to see improvements in the quality of the reconstructed 3D matrix and, thus, in the reprojected reference images. The precision of implemented registration algorithms for aligning the experimental projection images to the reprojected reference images becomes more important as the iteration continues. We have employed several registration algorithms in our approach including (1) reverse projection registration (RP) (Yang *et al.*, 2015 ▸), (2) the center of mass alignment (CM) (Azevedo *et al.*, 1990 ▸; Hogan *et al.*, 1993 ▸), (3) phase correlation alignment (PC) (Foroosh *et al.*, 2002 ▸), (4) the scale invariant feature transform (SIFT) (Lowe, 2004 ▸) and (5) the intensity-based automatic image registration (IAIR) available in Matlab’s image-processing toolbox (Mathworks, 2018 ▸).

Since the strength and the weakness of the registration algorithms are different, the sequence of executing algorithms should be carefully selected for different stages of the iterations. For example, the RP method is effective for determining the static offset of the rotational axis with respect to the center column of the projection images. It is, however, not capable of correcting the random image jitter. The RP method also relies on the availability of the projection images in reversed viewing angles, which could be limited by a missing wedge caused by the experimental geometry. The CM method is computationally efficient. It, unfortunately, has limited precision and is only applicable when the sample is smaller than the field-of-view (FOV) in the horizontal direction. The PC method shows a good balance between the efficiency and the precision, whereas SIFT and IAIR are both more computationally intensive but more precise. In our implementation, SIFT and IAIR correct both the translational and the rotation errors. One downside of the SIFT and IAIR methods is the weak robustness for the registration of lower-quality images.

**Figure 2:**
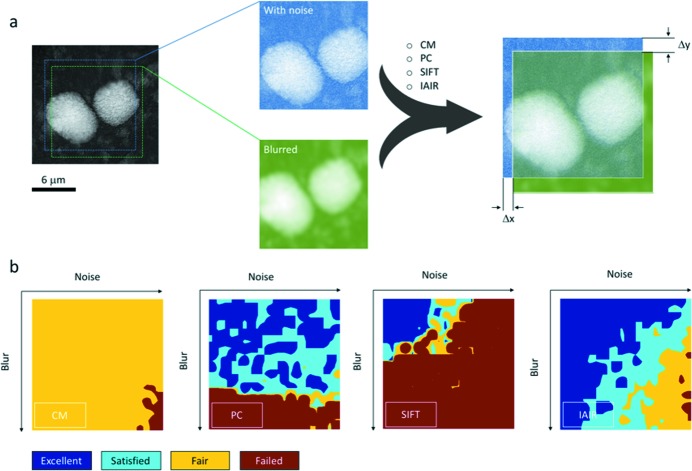
Evaluation of the precision and the robustness of different image registration algorithms with added imperfections. (*a*) Proposed simulation strategy: two sub-regions with known lateral offset are cropped from an identical image. After applying noise and/or blurring effects on the sub-regions, they are registered using four different algorithms. The calculated amounts of image offset (Δ*x* and Δ*y*) are then compared against the known true values. (*b*) The performance of different algorithms (from left to right: center of mass, phase correlation, scale invariant feature transform, and intensity-based automatic image registration) as a function of artificially induced noise and image blurring. Registration results with error smaller or equal to 1 pixel are labeled as ‘excellent’; results with error between 1 and 2 pixels are labeled as ‘satisfied’; results with error between 2 and 5 pixels are labeled as ‘fair’; results with error larger than 5 are considered as ‘failed’.

For a more thorough evaluation of the registration algorithms implemented in this work, we adopt the strategy of numerical simulation (Fig. 2 ▸). Two different sub-regions with known lateral offset are cropped from an arbitrarily selected raw projection image, which contains two particles of battery cathode materials. Different degrees of noise (including Poisson noise, Gaussian noise and salt & pepper noise) and blurring (Gaussian) are applied to these two sub-regions, respectively, before they are subjected to the image registration. This approach simulates the task of step *c* illustrated in Fig. 1 ▸. The calculated amount of offset (Δ*x* and Δ*y*) is compared against the known true values, and the errors are used to quantify the quality of the image registration algorithms. As shown in Fig. 2(*b*) ▸, although the CM method is very robust against the poor quality of the input images, its precision is certainly below satisfactory. The PC method is robust against the noise in the image, but its performance is not very stable as illustrated by the irregular pattern in the upper part of the corresponding map. SIFT is the least robust method, but it offers good precision when the input data are of good quality. IAIR, on the other hand, performs rather well although it is the most computationally intense method. Taking the characteristics of these registration algorithms into consideration, we have proposed a sequence (CM^5^–PC^15^–SIFT^10^–IAIR^20^) that shows the best performance in our test to be presented below.

**Figure 3:**
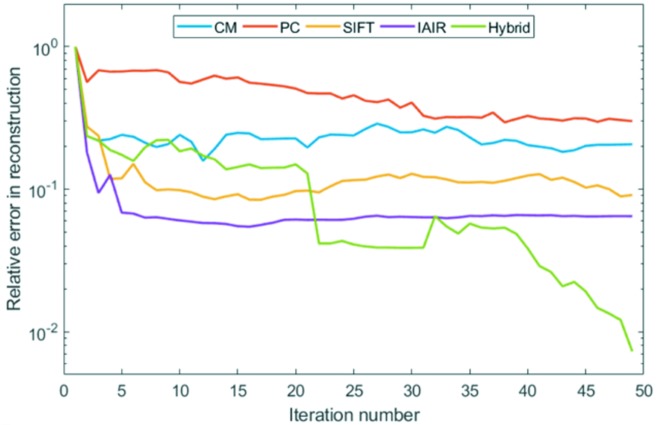
The quality of the tomographic reconstruction changes as a function of the iteration number as the dataset is going through the proposed projection image registration procedure.

In the experimental demonstration, we present the results acquired using the transmission X-ray microscope at beamline 6-2c of Stanford Synchrotron Radiation Lightsource (Andrews *et al.*, 2008 ▸), whose nominal spatial resolution is ∼30 nm. We conduct nano-tomographic investigation of the battery electrode’s secondary particles (LiNi_*x*_Mn_*y*_Co_*z*_O_2_, NMC). These particles are loaded into a quartz capillary of diameter 100 µm, which is significantly larger than the FOV (∼30 µm). For quantitative assessment of reconstruction quality, we first run the registration through many iterations until it reaches convergence regardless of which algorithm is used. We then use the reconstruction results as the ground truth and compare the reconstructed slices at each iteration against it by calculating the mean squared error and normalize it to the corresponding value at the initial iteration. The definition of the relative reconstruction error after ‘ite’ iterations (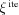) is shown in equation (1) for clarification. In equation (1), 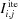 denotes the intensity at pixel (*i*, *j*) in the reconstructed result after ‘ite’ iterations. 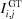 denotes the corresponding pixel intensity in the ‘ground truth image’,
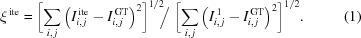
Fig. 3 ▸ shows the reduction of the relative reconstruction error as a function of the iteration number. It is clear that the performance of the image registration method is not optimal when any single registration algorithm is involved in the iterations. Although some of them rapidly improve the result in the first few iterations, they quickly reach a local minimum, which hinders further improvements. The sequence that combines multiple algorithms (the hybrid method), on the other hand, shows significant advantages in quality.

**Figure 4:**
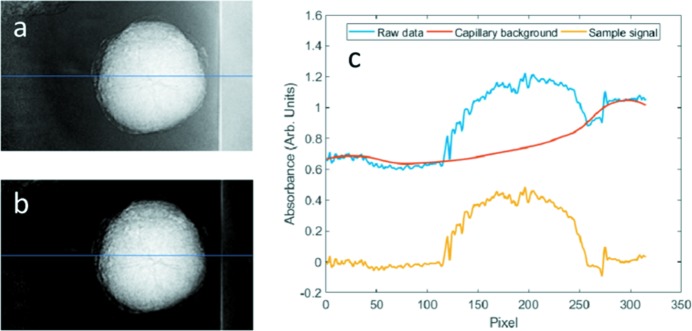
Background intensity removal before tomographic reconstruction. Panel (*a*) is the raw projection image at a certain angle. Panel (*b*) is the same image after background removal. Panel (*c*) shows a comparison of the intensity profile over the line highlighted in panels (*a*) and (*b*). Although the sharp edge of the capillary wall is still visible, after capillary background removal, the intensity profile over the area without sample of interest becomes flat.

It is worth mentioning that X-ray attenuation by the capillary’s absorption adds an intensity background to the projection images. This background also changes as a function of the viewing angle, complicating the image registration. For example, the CM method does not work in this scenario because the capillary background offsets the center of mass of the image significantly. As a result, background subtraction was performed prior to feeding the data into the image registration and reconstruction workflow. The projection images before and after the capillary background removal are shown in Fig. 4 ▸. The corresponding intensity line profile is shown in Fig. 4(*c*) ▸ to demonstrate the effectiveness of the capillary background removal.

**Figure 5:**
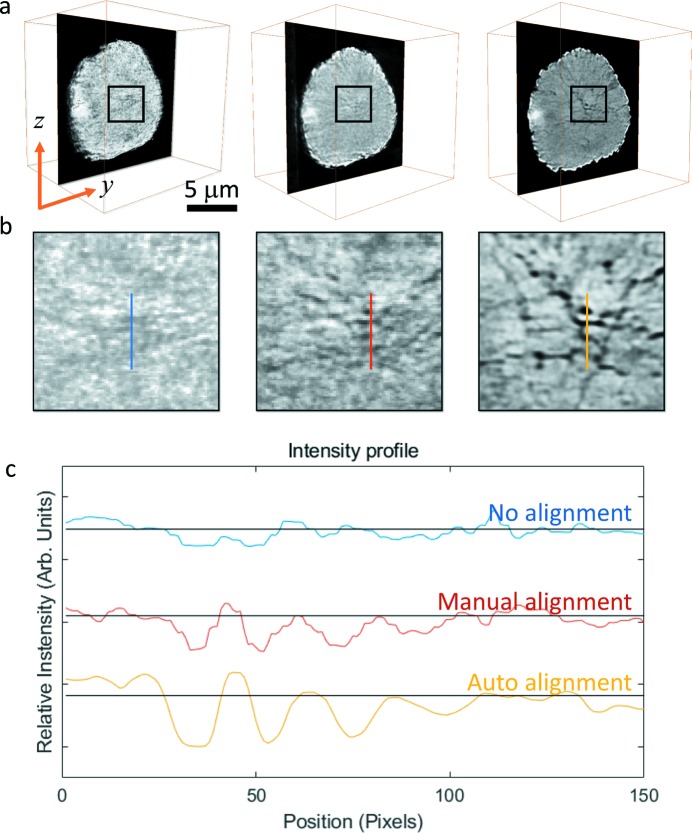
(*a*) Tomographic reconstruction results of projection data (left) without alignment, (middle) with manual alignment and (right) with automatic image registration developed in this work. The magnified view of the selected regions [marked as black squares in (*a*)] are shown in panel (*b*). (*c*) Intensity profiles over the lines highlighted in (*b*). The scale bar in panel (*a*) is 5 µm.

For further evaluation of the presented method, the 3D morpho­logical information of a substantially cycled battery cathode particle is shown in Fig. 5 ▸. The primary NMC particles of ∼200 nm agglomerate into secondary particles of ∼6 µm, which are mixed with conductive carbon and polymer binder and, then, casted onto the Al current collector. Upon electrochemical cycling, the NMC primary particles experience anisotropic volume expansion and contraction, which lead to a build-up of the mechanical strain within and between the primary particles. The accumulation of the mechanical strain eventually leads to the formation of cracks at the secondary particle level, reducing the internal ion and electron conductivity, which eventually translates into the degradation of the battery performance (Mu *et al.*, 2018 ▸; Liu *et al.*, 2017 ▸; Xia *et al.*, 2018 ▸; Ryu *et al.*, 2018 ▸). It is, therefore, of great interest to visualize and quantify the morphological defects, *i.e.* the cracks, in the secondary NMC particles that have gone through a different cycling history.

As a comparison between the reconstruction results with and without projection image registration, the virtual slices going through the particle’s center and the corresponding zoomed central area are shown in Figs. 5(*a*) and 5(*b*) ▸. After applying the iterative projection image registration with the proposed registration sequence, significant improvement in the quality of the tomographic reconstruction is clearly observed in the images, and is further confirmed by the corresponding line profiles shown in Fig. 5(*c*) ▸, which clearly demonstrates the enhanced contrast and the reduced noise. Interconnected crack structure is observed within the particle, highlighting the complicated chemomechanical interplay that occurred in the particle as it went through repeated electrochemical cycling (Xu *et al.*, 2018 ▸).

**Figure 6:**
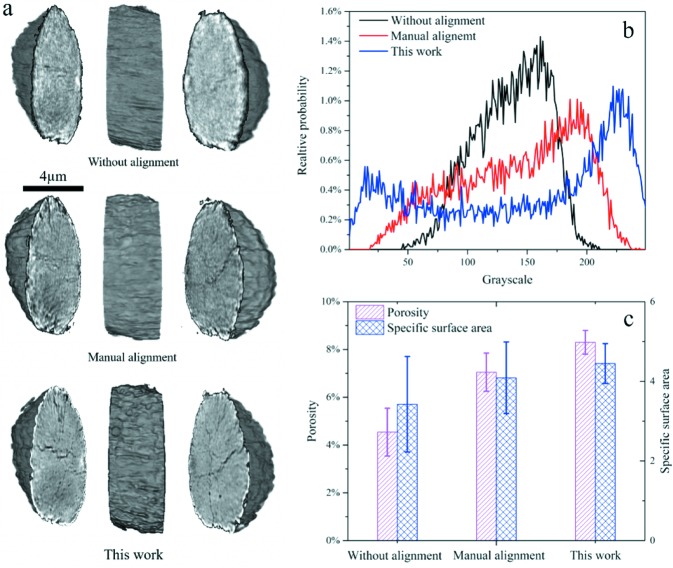
Visualization and quantification of the nanoscale X-ray tomographic data on a battery electrode particle that has gone through substantial cycling. (*a*) 3D rendering of the data without proper alignment (top), with manual alignment (middle), and with auto-alignment presented in this work (bottom). (*b*) Intensity histogram plot of the data shown in panel (*a*). (*c*) Comparison of the morphological quantification for the data, shown in panel (*a*), according to the registration methods. The scale bar in (*a*) is 4 µm.

While the visual assessment of the image quality is effective as human eyes are sensitive to the image artifacts and the noise, morphological quantification of the imaging data (Liu *et al.*, 2016 ▸) is also valuable because it can extract the structural information from the data with good consistency and automation in a statistically meaningful manner. In the tomographic study of the battery electrode particles, the information regarding the cracking induced porosity, surface area and morphological complexity is critical to understanding the mechanism of electrochemical degradation. We, therefore, show the 3D rendering and quantification of the reconstructed volume with and without the presented alignment procedure (Fig. 6 ▸). The presented automatic alignment method significantly enhances the image quality and reduces the artifacts. The improvement in the image contrast (Figs. 6*a* and 6*b* ▸) significantly promotes the accuracy in the image segmentation, which offers better fidelity in the quantification results (Fig. 6*c* ▸). The formation of the morphological defects in the particle, *i.e.* cracks, reduces the particle’s internal electric conductivity. The cracks also allow the liquid electrolyte to infiltrate into the particle, forming a new solid electrolyte interphase that alters the ionic diffusion pathways within the particle. The complicated interplay of the structural and chemical defects at the nanoscale could be responsible for the performance degradation and even the battery failure.

The above-discussed case study has nicely demonstrated the effectiveness and quality of the presented tomographic data registration method. In the following, we show the application of this method to the study of a more complicated sample, a small piece of shale rock with complicated internal structures. The study of shale is attracting global research interest due to the large amount of projected shale gas/oil production (US-EIA, 2018 ▸). The observation and understanding of the fine pore network in the shale rock could critically inform the design of the shale gas/oil extraction protocols, which could lead to better efficiency and less negative environmental impact.

**Figure 7:**
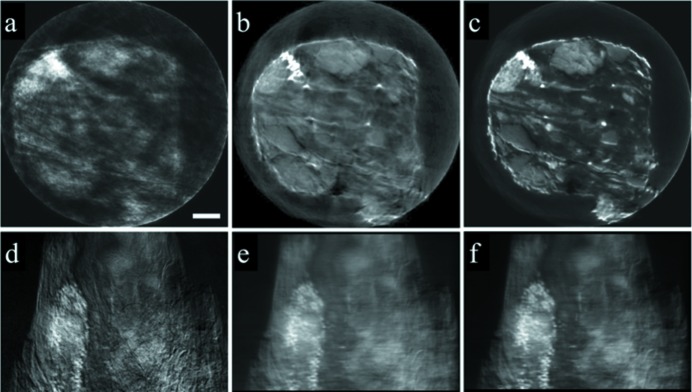
Reconstructed slices through the center of the shale sample without alignment (*a*) and with manual (*b*) or automatic (*c*) alignment. Panel (*d*) is the experimentally measured projection image. Panels (*e*) and (*f*) are the numerically reprojected images, calculated from the manual and auto-aligned 3D matrixes, respectively. The scale bar in panel (*a*) is 2 µm.

As presented in Fig. 7 ▸, the reconstructed slice with auto-alignment (Fig. 7*c* ▸) and that with manual alignment (Fig. 7*b* ▸) are both significantly better than the reconstruction result without alignment (Fig. 7*a* ▸). The automatic alignment once again demonstrates better performance, as shown by the superior image quality in Fig. 7(*c*) ▸. The numerically reprojected image from the auto-aligned 3D data (Fig. 7*f* ▸) is sharper than that calculated from the manually aligned 3D data (Fig. 7*e* ▸). The raw projection image at the same viewing angle (Fig. 7*d* ▸) is sharp and, however, rather noisy. The improvement in the signal-to-noise ratio in Fig. 7(*f*) ▸ (as shown by the fact that the pyrite particles are more clearly defined in Fig. 7*f* than that in Fig. 7*d* ▸) is achieved because the 3D result retains the contribution of projection images at different viewing angles. The reprojection of the 3D matrix is, thus, superior in its signal-to-noise ratio if the quality of the tomographic reconstruction is ensured. In this case study, our method is successfully applied to the study of a sample with much more complicated internal structure, highlighting the robustness of our method.

## 3. Conclusion   

The alignment of the projection images is a critical step that can dramatically affect the tomographic reconstruction. When imaging at nanoscale resolution, the alignment becomes non-trivial because the imperfections in the mechanical system become detectable in the imaging data. Random jitter in the projection images causes a severe point spread function and image artifacts that hinder the observation of fine morphological and chemical features at the nanoscale.

In this work, we developed an iterative image alignment method that involves several different image registration algorithms. A specific sequence was developed, showing the optimal performance in the presented case studies. We reconstructed the nanoscale X-ray tomographic data of a battery electrode particle that has gone through substantial electrochemical cycling. The herein-developed method successfully registered the projection images and reconstructed the 3D data with good fidelity, which facilitates more precise quantification of the particle’s morphology. The observed formation of the fine cracks in the battery electrode particle suggested that the interplay of the nanoscale morphological and the chemical defects are responsible for the particle degradation. To further evaluate the performance of our method, we present the application of our method in a study of a small piece of shale sample, which has a complicated internal structure. Our result shows significant improvement compared with manual alignment and highlights the accuracy and robustness of the algorithm. The developed method has been implemented in an in-house-developed software package known as *TXM-Wizard* (Liu *et al.*, 2012 ▸). We have also implemented a graphical user interface that allows the user to easily modify/optimize the iteration sequence for specific applications.

Finally, we point out here that, when conducting the nanoscale X-ray spectro-tomographic studies (Meirer *et al.*, 2011 ▸; Wei *et al.*, 2018 ▸), the registration of the projection images becomes even more difficult as the X-ray energy is also scanned in the experiment. The change of X-ray energy will result in a different absorption coefficient and different magnification in some cases. The projection image alignment method developed herein can be readily applied to the spectro-tomographic datasets thanks to the robustness of the proposed sequence.
